# Development, Characterization, and Immunomodulatory Evaluation of Carvacrol-loaded Nanoemulsion

**DOI:** 10.3390/molecules26133899

**Published:** 2021-06-25

**Authors:** Amanda Gabrielle Barros Dantas, Rafael Limongi de Souza, Anderson Rodrigues de Almeida, Francisco Humberto Xavier Júnior, Maira Galdino da Rocha Pitta, Moacyr Jesus Barreto de Melo Rêgo, Elquio Eleamen Oliveira

**Affiliations:** 1 Laboratory of Synthesis and Drug Delivery, State University of Paraiba, João Pessoa 58071-160, PB, Brazil; amandagabrielle6@gmail.com (A.G.B.D.); limongi.rafael@gmail.com (R.L.d.S.); 2 Laboratory of Immunomodulation and Novel Therapeutic Approaches, Federal University of Pernambuco, Recife 50670-901, PE, Brazil; andersonr.almeida@hotmail.com (A.R.d.A.); mgrpitta@gmail.com (M.G.d.R.P.); moacyr.rego@ufpe.br (M.J.B.d.M.R.); 3 Department of Pharmacy, Federal University of Paraíba, João Pessoa 58051-900, PB, Brazil; ffhxjunior@yahoo.com.br

**Keywords:** carvacrol, cytokine, immunoregulation, nanoemulsion

## Abstract

Carvacrol (CV) is an essential oil with numerous therapeutic properties, including immunomodulatory activity. However, this effect has not been studied in nanoemulsion systems. The objective of this study was to develop an innovative carvacrol-loaded nanoemulsion (CVNE) for immunomodulatory action. The developed CVNE comprised of 5% *w*/*w* oily phase (medium chain triglycerides + CV), 2% *w*/*w* surfactants (Tween 80^®^/Span 80^®^), and 93% *w*/*w* water, and was produced by ultrasonication. Dynamic light scattering over 90 days was used to characterize CVNE. Cytotoxic activity and quantification of cytokines were evaluated in peripheral blood mononuclear cell (PBMC) culture supernatants. CVNE achieved a drug loading of 4.29 mg/mL, droplet size of 165.70 ± 0.46 nm, polydispersity index of 0.14 ± 0.03, zeta potential of −10.25 ± 0.52 mV, and good stability for 90 days. CVNE showed no cytotoxicity at concentrations up to 200 µM in PBMCs. CV diminished the production of IL-2 in the PBMC supernatant. However, CVNE reduced the levels of the pro-inflammatory cytokines IL-2, IL-17, and IFN-γ at 50 µM. In conclusion, a stable CVNE was produced, which improved the CV immunomodulatory activity in PBMCs.

## 1. Introduction

Medicinal plants can be a source of bioprospection for new bioactive products with commercial interest, including essential oils extracted from a wide variety of plants [1]. The biological activities of essential oils or isolated compounds obtained from the genus *Origanum* (*Origanum vulgare*, *O. compactum*, *O. dictammus*, *O. onites*, and other species) have been studied [2,3]. Carvacrol (CV) is the main monoterpenic phenol compound isolated from *O. vulgare* (oregano) essential oil [4,5]. CV exhibited anticancerous, antioxidant, anti-helminthic, antidepressant, antinociceptive, and antimicrobial activities [6,7]. In addition, carvacrol has been described as a potential immunomodulator of the anti-inflammatory response [8,9].

CV has anti-inflammatory properties due to its interference in the arachidonic acid cascade that inhibits the production of prostaglandins, an important agent that stimulates inflammatory responses [10]. CV has been reported to inhibit the activity of cyclooxygenase (COX), such as COX-1, which promotes anti-inflammatory and antithrombotic effects [11], and COX-2, which inhibits prostaglandin biosynthesis [12]. Furthermore, CV has been demonstrated to inhibit inflammatory cytokines, such as tumor necrosis factor (TNF)-α, interleukin (IL)-6, interferon-gamma (IFN)-γ, IL-17, and IL-1β [13,14]. In addition, this compound also showed the improvement in anti-inflammatory mediators, increasing the levels of transforming growth factor (TGF)-β and IL-10 [15]. Despite their biological activities, nanosystems containing CV have not yet been produced for immunomodulatory purposes.

Nanoemulsions are colloidal dispersions of two immiscible liquids stabilized by surfactants with small droplet sizes [16,17]. The advantages of this system include the enhancement of the solubility and bioavailability of lipophilic molecules (e.g., CV), controlled drug release, and protection from enzymatic degradation [18,19]. Nanoemulsions can be produced using different methodologies, including high-energy methods such as high-pressure homogenization, ultrasonication, and microfluidization [20,21,22,23].

Therefore, the aim of this study was to evaluate the immunomodulatory activity of carvacrol-loaded nanoemulsions (CVNEs). To achieve this objective, oil/water (*o*/*w*) nanoemulsions were produced, and their stability was analyzed over 90 days. Subsequently, cytotoxicity and immunomodulatory activity of CVNE were evaluated in peripheral blood mononuclear cells (PBMCs).

## 2. Results and Discussion

### 2.1. Characterization and Stability Study of the Nanoemulsions

**Figure 1:**
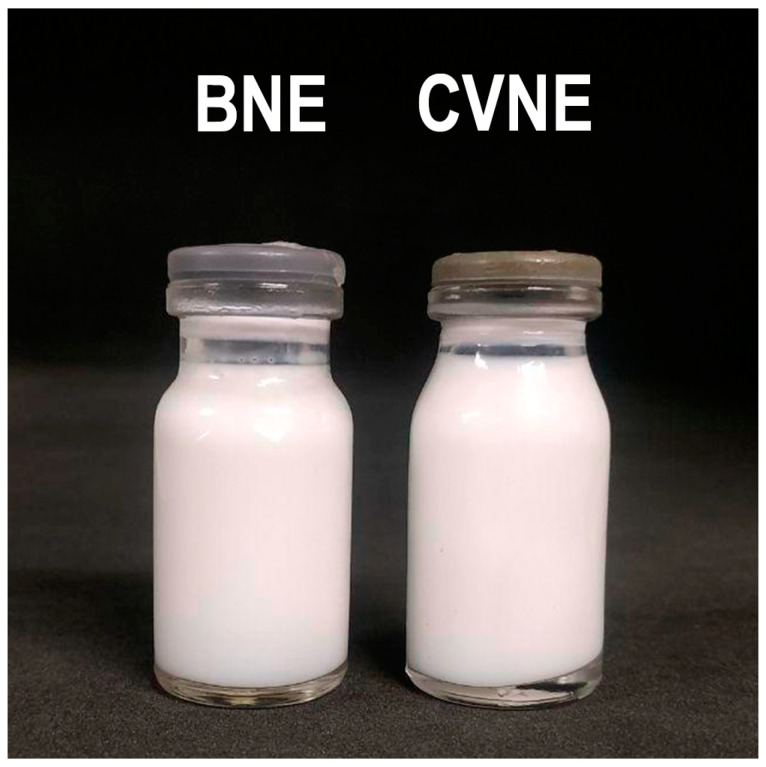
Visual aspect of unloaded nanoemulsion (BNE) and carvacrol loaded nanoemulsion (CVNE) after 90 days of storage at 4 °C.

In this study, a CVNE and an unloaded nanoemulsion (blank formulation, BNE) were produced using ultrasonication. Previously, to assess the possible instability of nanoemulsions, the CVNE and the BNE were submitted to accelerated stability studies. Mechanical stress and thermal stability tests can predict important information regarding the stability of the formulation. When samples are subjected to increased temperature and centrifugation forces, the Brownian movements are increased, which allows the dispersed droplets to approach and increase the probability of collision [24]. Collision between these droplets can promote aggregation, increasing droplet size and coalescence rate. CVNE and BNE did not show alterations in the appearance (creaming or phase separation) after centrifugation or heating. Furthermore, even after 90 days of formulation, the nanoemulsions proved to be macroscopically stable and preserving the milky appearance without creaming of phase separation (Figure 1). The use of nonionic surfactants and the small size of the droplets may have favored short-range repulsive forces, such as steric overlap, hydration, and thermal fluctuation interactions [25], preventing destabilization.

The mean droplet diameter, polydispersity index, and zeta potential of CVNE and BNE were evaluated by dynamic light scattering technique for 90 days and are shown in Table 1 and Table 2, respectively. The droplet size of a nanoemulsion results from the processing conditions employed, the interactions of the components, and the adsorption of the surfactants with the oil phase [26]. Ultrasonication was chosen as the technique to develop the nanoemulsions as it favors the obtention of small drops due to the formation of different disruptive forces caused by the sonicator, causing turbulence and cavitation in the sample [27,28]. This technique requires a low concentration of surfactant, low cost of production, and greater ease of operation and cleaning [29].

To quantify the amount of CV present in the nanoemulsion, the UV-vis spectrum was used. Data were calculated using the absorbance of the CVNE dilution (A = 0.330 ± 0.004) on the standard curve (y = 0.01646x − 0.02384) with a coefficient of determination of 0.9992. CVNE shows a drug recovery of 83.97% ± 1.02 and a drug loading content of 4.29 mg/mL of CV. Hussein et al. showed a drug recovery of 49.3% with nanoemulsions containing CV developed by high pressure homogenization with 1% surfactant [30]. Additionally, Khan et al. obtained a drug recovery close to 80% of CV in a nanoemulsion containing MCT and polysorbate 80 using the method of ultrasonication and high pressure homogenization [31]. Another important parameter that must be evaluated is the pH of the nanoemulsions. It was observed a pH variation from 5.6 to 5.85 in the CVNE, and a variation from 5.62 to 5.88 in the BNE during the 90 days of analysis. The slightly acidic pH of CVNE does not have many clinical implications in the gastric environment [32].

The CVNE presented a mean droplet diameter (z-average) of 169.06 ± 1.10 nm after 90 days of preparation, with a slight variation when compared with the mean droplet size on day 1, confirming the good stability of the nanoemulsion (for more details, see Supplementary Figures S1 and S2). Understanding the stability of nanoemulsions over time is an important parameter for biological studies. The droplet size stability of CVNE can be attributed to the selection of the surfactants, which present the hydrophile–lipophile balance (HLB) value close to the required HLB of the oil phase and the presence of medium-chain triglycerides (MCTs) [23,26,27]. MCT was selected because it is a good Ostwald ripening inhibitor and contributes to the production of nanoemulsions with small droplet sizes [33,34].

The BNE shows a mean droplet diameter close to 124 nm, with no significant changes over 90 days (Table 2). On comparing the mean droplet sizes of CVNE and BNE, an increase in the droplet size of CVNE was observed. The oil phase of the CVNE consists of a mixture of CV and MCT. The interaction of MCT and CV in the oil phase of CVNE may have contributed to the increase in droplet size in relation to the BNE. It has already been reported that the droplet size of CVNEs can vary according to the proportion of CV/MCT ratio in the oil phase [35]. The proportions of CV/MCT, surfactants, and ultrasonic parameters were responsible for the size of the CVNE. Recent studies carried out by Felício et al. and Mazerei et al. with carvacrol have also obtained similar droplet sizes using CV in nanoemulsions containing Tween^®^ 80 and Span^®^ 80 as surfactants, with size droplets varying between 125 and 164 nm and 148 and 151 nm, respectively [20,36].

Other parameter used to evaluate the stability of the nanoemulsions was the polydispersity index (PDI). The term “polydispersity” is used to describe the degree of uniformity of the size distribution of the droplets within a system [37]. Moreover, PDI can be evaluated with respect to time. If variations in PDI values are observed, it may indicate the destabilization of the formulation due to phenomena such as coalescence or droplet aggregation. On day 1, CVNE exhibited a PDI of 0.14 ± 0.03, and at the end of the evaluation at day 90, there was no statistical change in the PDI (*p* > 0.05). Nanoemulsions containing relatively small droplets (<200 nm) with PDI values < 0.3 are considered monodisperse populations [38,39]. Thus, the CVNE and BNE were considered monodisperse according to the parameters described above. Another study on CVNEs containing MCT exhibited similar results [35].

Another important stability parameter to be observed is the zeta potential of the nanoemulsions. Two nonionic surfactants were used in the development of nanoemulsions. It was observed that CVNE and BNE present slightly negative zeta potentials (−10 to −15 mV and −13 to −18 mV, respectively). The more positive or negative the zeta potential, the better the nanoemulsion stability [39]. These values can be attributed to the presence of anions (such as OH^−^) released by the oil components and surfactants [40,41]. Another study of CVNEs containing MCT and a nonionic surfactant also showed similar zeta potential values [24].

### 2.2. Cytotoxicity Activity

The in vitro cytotoxicity of CVNE, BNE, and CV was evaluated in PBMCs using the MTT [3-(4,5-dimethylthiazol-2-yl)-2,5-diphenyltetrazolium bromide] assay.

CVNE and BNE showed an average cell viability greater than 95% at all tested concentrations (50 µM, 100 µM, and 200 µM). CV showed an average cell viability greater than 99% at a concentration of 50 µM, and greater than 90% at concentrations of 100 and 200 µM. Based on these results, concentrations of 25 and 50 µM were selected for assays of immunomodulatory activity.

### 2.3. In Vitro Cytokine Evaluation

Inflammation is a key response that involves the participation of a variety of chemical mediators and signaling pathways involved in tissue repair. The inflammatory process can be characterized by the activation of cascades of mediators that regulate important mechanisms of the inflammatory response, such as vascular permeability, recruitment of leukocytes in the blood, and cytokine production [42,43]. The uncontrolled production of these immunomodulators can cause pain, damage to the injured tissue, and loss of local function [42,44]. Herein, PBMCs were used to evaluate the levels of cytokines in vitro, as they can serve as a model for understanding cytokine expression [45].

PBMCs consist of a group of cells, including lymphocytes and monocytes/macrophages, which play an important role in the inflammatory response [46]. Thus, these cells can be used in in vitro models to quantify the cytokines involved in inflammatory processes [47,48]. However, it is important to note that our study is mainly concerned with the cytokines produced by T cells due to the in vitro stimulus used. Systemic inflammation can be characterized by high levels of inflammatory biomarkers in the bloodstream produced by PBMCs, such as TNF-α, IL-1β, IL-6, and other cytokines [45]. Bioactives that show a reduction in the production of pro-inflammatory cytokines are good candidates for anti-inflammatory drugs.

**Figure 2:**
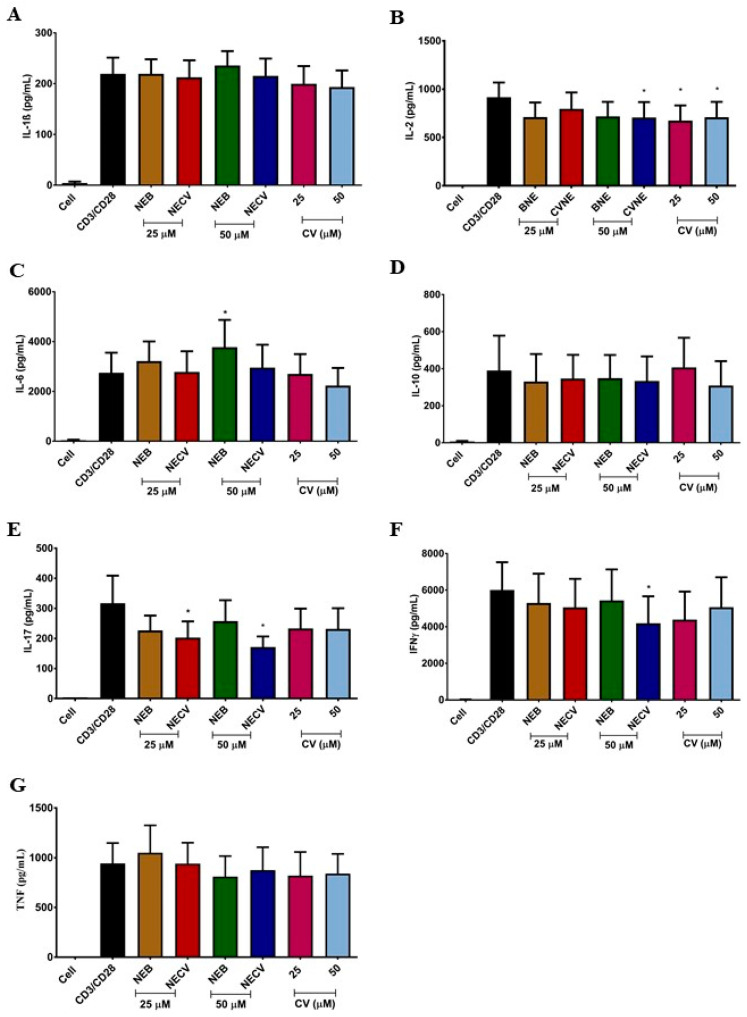
Immunomodulatory activity of carvacrol-loaded nanoemulsion (CVNE), blank nanoemulsion (BNE), and carvacrol (CV) in peripheral blood mononuclear cells. The bars represent cytokine levels: IL-1β (**A**); IL-2 (**B**); IL-6 (**C**); IL-10 (**D**); IL-17 (**E**); IFN-γ (**F**); and TNF-α (**G**) at 25 and 50 µM; non-stimulated (Cell); and stimulated cell (anti-CD3 and anti-CD28 antibodies) conditions. * *p* < 0.05 (Mean ± SEM, followed by Wilcoxon’s signed rank test).

The levels of immunosuppressive cytokines (IL-10), inflammatory mediators (IL-6), and proinflammatory cytokines (IL-1β, IL-2, IL-17, IFN-γ, and TNF-α) were quantified in PBMCs of healthy individuals stimulated with anti-CD3 and anti-CD28 antibodies (Figure 2).

CV (25 and 50 µM) reduced (*p* = 0.007 and *p* = 0.023) the levels of IL-2 (median [min–max] 661.4 [104.6–1277] pg/mL and 740.9 [82.88–1286] pg/mL, respectively) when compared to the stimulus condition with anti-CD3 and CD-28 (1051 [202.7–1331] pg/mL) (Figure 2) in the PBMC culture supernatant. In vivo assays have shown that CV reduces the levels of pro-inflammatory cytokines in autoimmune encephalomyelitis and asthma models [15,24,42]. Treatment in a clinical trial with CV for two months reduced the levels of inflammatory cytokines and increased the levels of anti-inflammatory cytokines [49]. Other studies have described the activity of CV, or essential oil containing CV, to decrease the levels of pro-inflammatory cytokines and inflammatory biomarkers [13,45,46,47,48]. Despite these, other results confirmed the influence of CV on IL-2 levels, and these studies also observed the modulation of more cytokines, which may be associated with the methodology used in each experiment. In in vivo tests, other biological components and modulators can directly or indirectly interfere with the inflammatory response in the cytokine cascade.

The CVNE formulation at 25 µM reduced IL-17 levels (*p* = 0.015) (165.4 [54.08–454] pg/mL) when compared to the anti-CD3 and anti-CD-28 condition (235.4 [83.67–728.7] pg/mL). At a concentration of 50 µM, the CVNE formulation reduced the levels of IL-2 (*p* = 0.015) (746.8 [126.4–1322] pg/mL), IFN-γ (*p* = 0.015) (2583 [69.44–11,419] pg/mL), and IL-17 (*p* = 0.031) (142 [49.50–325.4] pg/mL) (Figure 1) when compared to the stimulus condition with anti-CD3 and anti-CD28 antibodies (1051 [202.7–1331], 5221 [29.44–11,906] and 235.4 [83.67–728.7] pg/mL, respectively). This increase in cytokines modulated by CVNE in relation to CV can be attributed to the size of the nanoemulsion, which increases the contact surface and biological interaction [50]. IL-17 plays an important role in inducing and maintaining chronic inflammation by stimulating other cells to secrete more cytokines and growth factors [51]. In addition, IL-17 and IFN-γ play fundamental roles in the development and aggravation of autoimmune diseases [15]. IFN-γ triggers M1 macrophages and induces the synthesis of reactive oxygen species (ROS) and reactive nitrogen species (NOS) by antigen-presenting cells [15]. These findings offer good results for in vivo tests. Some inflammatory pathologies present high levels of these cytokines, such as systemic sclerosis, multiple sclerosis, and asthma [15,41,45].

BNE was formulated without the bioactive compound to analyze whether the constituents of the formulation could be toxic or possibly interfere with the production of cytokines. It was observed that BNE did not show cytotoxic activity and did not interfere with cytokine production, except for an increase (*p* = 0.039) in IL-6 expression. The BNE oil phase was composed of MCT. MCT can be mixed with other oils because it has no biological activity. In addition, two non-ionic surfactants, Span 80 and Tween 80, were used in the aqueous phase. These surfactants were chosen because of their known lack of biological toxicity [52,53]. The results obtained by BNE are significant, emphasizing that the cytokine inhibitory activity of CVNE was attributable to the properties of the CV.

In conclusion, we observed that it was possible to formulate a stable nanoemulsion with CV over 90 days of evaluation with 83.97% ± 1.02 of encapsulation efficiency. CVNE showed no cytotoxic activity at the concentrations evaluated in this study. In general, CVNE improved the biological activity of CV by inhibiting the levels of pro-inflammatory cytokines (IL-2, IL-17, and IFN-γ) at 50 µM in PBMC culture supernatant. In this way, we were able to obtain an immunomodulatory potential, and we suggest that new studies be carried out, such as in vivo tests, to better understand the performance of CVNE.

## 3. Materials and Methods

### 3.1. Materials

CV oil (5-isopropryl-2-methylphenol, purity 98%) and sorbitan monostearate 80 (Span 80^®^) were purchased from Sigma-Aldrich (São Paulo, Brazil). Polysorbate 80 (Tween 80^®^) was provided by Vetec Química Fina LTDA (Duque de Caxias, Rio de Janeiro, Brazil). MCTs (Mygliol 812^®^) were acquired from Sasol (Brunsbüttel, Germany). Ethanol was sourced from Merck (São Paulo, Brazil), and dimethyl sulfoxide (DMSO) was purchased from NEON (Suzano, São Paulo, Brazil). The anti-CD3 and anti-CD28 antibodies were purchased from Ebiosciences (San Diego, CA, USA), Ficoll Paque Plus was purchased from GE Healthcare Biosciences (Pittsburgh, PA, USA), and the media were purchased from Gibco (Thermo Fisher Scientific, Waltham, MA, USA). The human ELISA kits were provided by BD Biosciences (USA, for INF-γ, TNF-α, IL-6, and IL-10), eBioscience (USA, for IL-2 and IL-17), and Invitrogen (USA for IL-1β).

### 3.2. Preparation of Oil-in-Water Nanoemulsions

To prepare the NECV, initially, all components of the oily phase were weighed (0.05 g CV + 0.45 g MCT). After that, the surfactants (0.12 g Tween 80^®^ + 0.08 g Span 80^®^) were added to the oily phase. Finally, distilled water (9.3 g) was added. In the BNE, CV was replaced with MCT. The CVNE was prepared by ultrasonication, and the components were subjected to four homogenization cycles in a sonication apparatus (model QE200, Ultronique, Brazil) for 1 min at 300 W using an ice bath, followed by an ultrasonic bath for 1 min [20].

### 3.3. Carvacrol Content

The CV content in the nanoemulsions was determined by ultraviolet–visible spectrophotometry (Genesys 10S UV-Vis, Thermo Fisher Scientific, Dreieich, Germany). Initially, the maximum absorption wavelength of CV was determined to be 276 nm in methanol by scanning UV-Vis spectroscopy ranging from 200 to 900 nm. The concentration of CV in the nanoemulsion was calculated using a calibration curve ((*n* = 7) y = 0.01646x − 0.02384; R^²^ = 0.9992). The CVNE was diluted in methanol (1:2000) and filtered using a 0.2 μm membrane filter (Kasvi, Brazil) [12]. All measurements were performed in triplicates. Drug loading content and drug recovery were calculated according to Equations (1) and (2), respectively.

(1) $$Drug\;loading\;content\;\left(\frac{mg}{mL}\right)=\frac{Amount\;of\;CV\;in\;the\;NECV}{Total\;amount\;of\;NECV}\;$$

(2) $$Drug\;recovery\;\%\;\left(\frac{w}{V}\right)=\left(\frac{Amount\;of\;CV\;in\;the\;NECV}{Amount\;of\;CV\;added}\right)\;\times \;100$$

### 3.4. Particle Size, Polydispersity Index and Zeta Potential

Particle size, PDI and zeta potential were determined using a Zetasizer instrument (Nano ZS, Malvern Instruments, Great Malvern, UK) using the dynamic light scattering technique at 25 °C. Measurements of mean droplet (Z-average) diameter of the intensity size distribution were carried out at a fixed scattering angle of 173°. The zeta potential was evaluated from the electrophoretic mobility under an electric field using the same equipment. Prior to measurement, the nanoemulsions were diluted with deionized water (1:25, *v*/*v*). All measurements were performed in triplicate, and the results are presented as a mean with standard deviation.

### 3.5. Accelerated Stability Tests

Centrifugation test was done by adding 5g of nanoemulsion to a centrifuge tube and loaded into a centrifuge (CP100NX, Hitachi, Japan). The emulsion was then subjected to centrifugal acceleration at 3000 rpm for 30 min at 25 ± 2 °C [54].

In thermal stress test, the nanoemulsion was gradually heated from 40 up to 80 °C, rising the temperature in intervals of 5 °C, keeping each temperature for 30 min [55]. After centrifugation or temperature test, the nanoemulsions were evaluated for the occurrence of phase separation. These assays were realized in triplicate.

### 3.6. Stability Study

Stability studies were carried out using CVNE and BNE over a period of 90 days. CVNE and BNE were kept in 10mL glass vials sealed with plastic caps and kept in a refrigerator at 4 ± 2 °C. The sampling time points were 1, 7, 20, 30, 60, and 90 days. The formulations were evaluated for changes in particle size, PDI, zeta potential, pH, and macroscopic aspect.

The pH of the CVNE and BNE was measured using a multipurpose autotitrator (model MPA-210, Tecnopon Instruments, Brazil) previously calibrated with buffer solutions of pH 4.0 and 7.0.

For the macroscopic aspect, color, appearance of the nanoemulsion, and phase separation were assessed.

### 3.7. Peripheral Blood Mononuclear Cells Isolation and Culture

PBMCs were isolated from the blood of healthy individuals after collection in heparinized tubes. The blood was centrifuged in Ficoll Paque Plus and PBMCs were separated. After separation, cells were counted and plated in 24-well plates (1 × 10^6^ cells/well) in RPMI-1640 (Gibco) medium supplemented with L-glutamine, 10% fetal bovine serum (FBS) (Gibco), 10 mM HEPES (4-(2-hydroxyethyl)-1-piperazineethanesulfonic acid), and 200 U/mL penicillin/streptomycin (Gibco). Subsequently, the cells were stimulated with anti-CD3 and anti-CD28 antibodies and treated with CV, CVNE, or BNE at concentrations of 25 µM and 50 µM of CV. Eight healthy individuals were included in this study. Exclusion criteria for controls were regular use of medications, chronic inflammatory disease, or alcohol or cigarette consumption in the last 15 days. The protocol was approved by the ethics committee of Universidade Federal de Pernambuco (CEP/CCS/UFPE/CAAE Nº 63517517.1.0000.5208).

### 3.8. In Vitro Cytotoxicity

The MTT method was used to evaluate the cytotoxic activity of the nanoemulsions and CV oil in PBMCs at concentrations of 50 µM, 100 µM, and 200 µM. The cells were plated in 96-well plates and exposed to different conditions for 48 h. Subsequently, 20 µL MTT (0.5 mg/mL) was added to each well of the plate, followed by the addition of 130 µL of 20% SDS after 3 h. Cell viability was assessed by the ability of cells to reduce MTT to formazan blue. The absorbance was measured 24 h later at a wavelength of 570 nm using a microplate reader (Elx808, Biotek, Winooski, VT, USA). Four independent experiments were conducted.

### 3.9. Cytokine Quantification

The cytokines IL-2, IL-6, IL-10, IL-17, IFN-γ, TNF-α, and IL-1β were quantified in the supernatant of PBMC cultures. Cytokine levels were quantified using human ELISA kits (BD Biosciences, San Jose, CA, USA) for IFN-γ, TNF-α, IL-6, and IL-10, eBioscience, San Diego, CA, USA for IL-2 and IL-17, and Invitrogen, Carlsbad, CA, USA for IL-1β. The lower detection limits for IL-1β, IFN- γ, TNF-α, IL-2, IL-17, IL-6, and IL-10 were 2.0 pg/mL, 4.7 pg/mL, 7.8 pg/mL, 2.0 pg/mL, 4.0 pg/mL, 4.7 pg/mL, and 7.8 pg/mL, respectively. All tests were performed according to the manufacturer’s recommendations.

### 3.10. Statistical Analysis

GraphPad Prism 6.0 software (GraphPad Software Inc., San Diego, CA, USA) was used for data analysis. In the stability study, one-way analysis of variance (ANOVA) was applied. In the in vitro study, the differences between conditions were calculated using Wilcoxon’s signed classification test. The results of continuous variables are expressed as mean ± standard error of the mean. The differences were considered significant when *p* < 0.05.

## Figures and Tables

**Table 1:** Parameters of stability: mean droplet diameter, zeta potential, polydispersity index, and pH of nanoemulsion with carvacrol (CVNE) over 90 days.

Parameters of Stability/Day Analysis	Mean Droplet Diameter (nm)	Polydispersity Index	Zeta Potential (mV)	pH
D1	165.70 ± 0.46	0.14 ± 0.03	−10.25 ± 0.52	5.6
D7	163.56 ± 0.60	0.14 ± 0.03	ND	5.7
D20	166.56 ± 1.22	0.16 ± 0.01	ND	5.94
D30	186.03 ± 1.27 *	0.25 ± 0.01	−10.83 ± 0.42	5.87
D60	164.43 ± 0.81	0.14 ± 0.01	−14.60 ± 0.44 *	5.85
D90	169.06 ± 1.10 *	0.14 ± 0.00	−14.67 ± 0.55 *	5.85

D1, day one; D7, day seven; D20, day 20; D30, day 30; D60, day 60; D90, day 90. * *p* < 0.05. ND: no determined.

**Table 2:** Parameters of stability: mean droplet diameter, zeta potential, polydispersity index, and pH of blank nanoemulsion (BNE) over 90 days.

Parameters of Stability/Day Analysis	Mean Droplet Diameter (nm)	Polydispersity Index	Zeta Potential (mV)	pH
D1	123.67 ± 1.24	0.18 ± 0.02	−13.00 ± 0.66	5.62
D7	121.87 ± 0.59 *	0.18 ± 0.01	ND	5.78
D20	128.27 ± 0.50 *	0.22 ± 0.01	ND	5.97
D30	128.23 ± 0.68 *	0.22 ± 0.00	−12.63 ± 0.38	5.75
D60	127.73 ± 0.06 *	0.19 ± 0.02	−16.93 ± 0.55 *	5.90
D90	124.47 ± 0.45	0.18 ± 0.01	−17.8 ± 0.00 *	5.88

D1, day one; D7, day seven; D20, day 20; D30, day 30; D60, day 60; D90, day 90. * *p* < 0.05. ND: no determined.

## Data Availability

The data presented in this study are available in supplementary material.

## Supplementary Materials

The following are available online. Figure S1: Size distribution by intensity of carvacrol loaded nanoemulsion (CVNE) in Day 1; Figure S2: Size distribution by intensity of unloaded nanoemulsion (BNE) in Day 1.

## Sample Availability

Samples of the nanoemulsions CVNE and BNE are available from the authors.
