# Supplementary material for: Development, Characterization, and Immunomodulatory Evaluation of Carvacrol-loaded Nanoemulsion
Source: Molecules. 2021 Jun 25;26(13):3899. doi: 10.3390/molecules26133899 (PMC8271444; doi:10.3390/molecules26133899)
Supplement: Supplementary file 1 [file molecules-26-03899-s001.zip › molecules-1246025-supplementary.pdf]

SUPPLEMENTARY MATERIALS

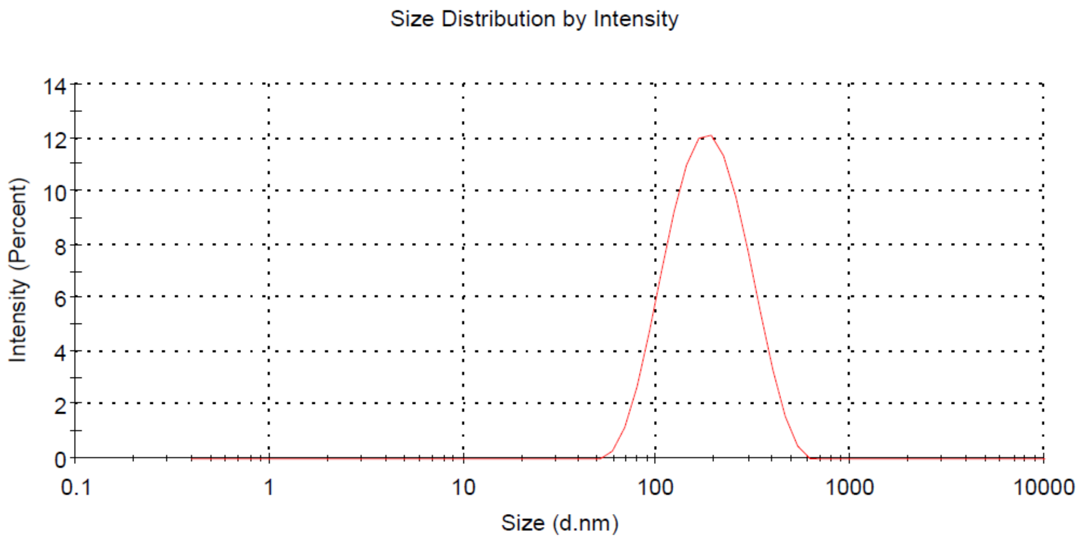

Figure S1. Size distribution by intensity of carvacrol loaded nanoemulsion (CVNE) in Day 1.

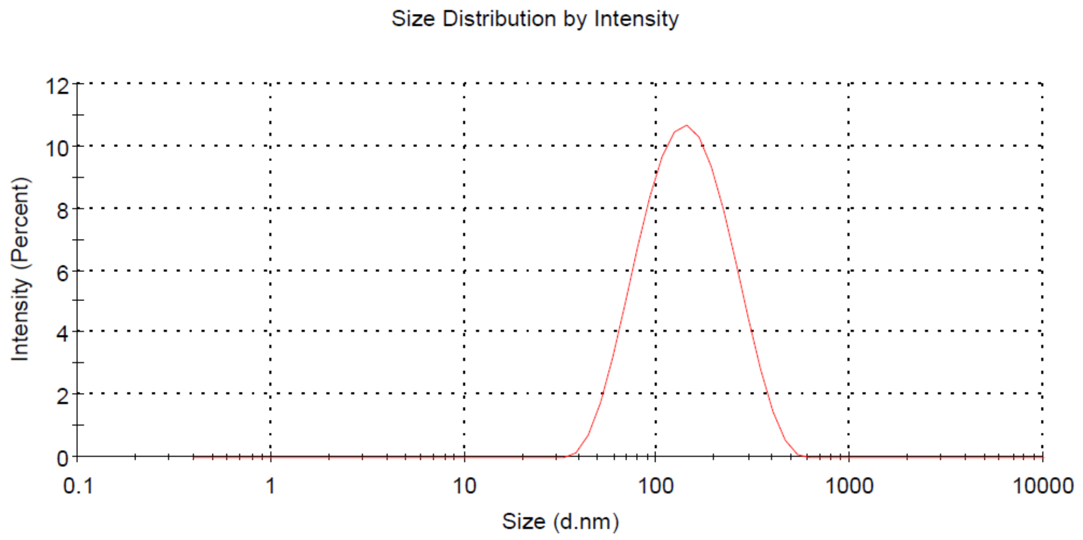

Figure S2. Size distribution by intensity of unloaded nanoemulsion (BNE) in Day 1.
